# Developmental Functions of the Dynamic DNA Methylome and Hydroxymethylome in the Mouse and Zebrafish: Similarities and Differences

**DOI:** 10.3389/fcell.2018.00027

**Published:** 2018-03-20

**Authors:** Peter Jessop, Alexey Ruzov, Martin Gering

**Affiliations:** ^1^School of Life Sciences, Queen's Medical Centre, University of Nottingham, Nottingham, United Kingdom; ^2^Division of Cancer and Stem Cells, Centre for Biomolecular Sciences, School of Medicine, University of Nottingham, Nottingham, United Kingdom

**Keywords:** DNA methylation, epigenetics, 5mC, 5hmC, TET, zebrafish, mouse, gene expression

## Abstract

5-methylcytosine (5mC) is the best understood DNA modification and is generally believed to be associated with repression of gene expression. Over the last decade, sequentially oxidized forms of 5mC (oxi-mCs) have been discovered within the genomes of vertebrates. Their discovery was accompanied by that of the ten-eleven translocation (TET) methylcytosine dioxygenases, the enzymes that catalyze the formation of the oxi-mCs. Although a number of studies performed on different vertebrate models and embryonic stem cells demonstrated that both TET enzymes and oxi-mCs are likely to be important for several developmental processes it is currently unclear whether their developmental roles are conserved among vertebrates. Here, we summarize recent developments in this field suggesting that biological roles of TETs/oxi-mCs may significantly differ between mice and zebrafish. Thus, although the role of TET proteins in late organogenesis has been documented for both these systems; unlike in mice the enzymatic oxidation of 5mC does not seem to be involved in zygotic reprogramming or gastrulation in zebrafish. Our analysis may provide an insight into the general principles of epigenetic regulation of animal development and cellular differentiation.

## DNA methylation and plasticity of gene expression in embryonic development

Embryonic development and organogenesis are epigenetic processes directed by intricate networks of transcriptional regulators (Boyer et al., 2005; Chambers et al., 2007). During embryogenesis, the transcriptional programmes of individual cells are influenced by cell intrinsic factors and stochastic events (Tang et al., 2011; Moris et al., 2016). Cells also need to be responsive to molecular signals originating from their microenvironment (Rossant and Tam, 2009; Shi and Wu, 2009). The plasticity of gene expression allows distinct populations of cells to arise within the embryo (Tam and Loebel, 2007). The regulation of gene expression during development is remarkably complex and heavily reliant upon epigenetic mechanisms, such as histone and DNA modifications (Reik, 2007).

Eukaryotic DNA can be methylated at cytosine residues, by DNA methyltransferases (DNMTs) (Bird, 2002). The methylation of a gene's cis-regulatory elements is generally considered to inhibit its transcription (Suzuki and Bird, 2008) and, remarkably, the impairment of DNA methylation regulation has been implicated in the pathologies of many developmental diseases (Robertson, 2005). Most metazoan DNA methylation (99.98% in human somatic cells, Jang et al., 2017) takes place at CpG dinucleotide sites within the genome. CpG sites represent approximately 7% of the mouse genome (Smith et al., 2009) and 5.3% of the zebrafish genome (Chatterjee et al., 2013). Regions of the genome with high CpG density, so-called CpG islands, generally overlap with mapped transcription start sites of identified gene promoters (Carninci et al., 2006; Potok et al., 2013). Importantly, DNA methylation is reversible (Wu and Zhang, 2014) and 5mC deposition and removal pathways operate during several key stages of vertebrate embryogenesis (Smith and Meissner, 2013).

## The TET enzymes and 5mC oxidized derivatives (oxi-mCs)

**Figure 1:**
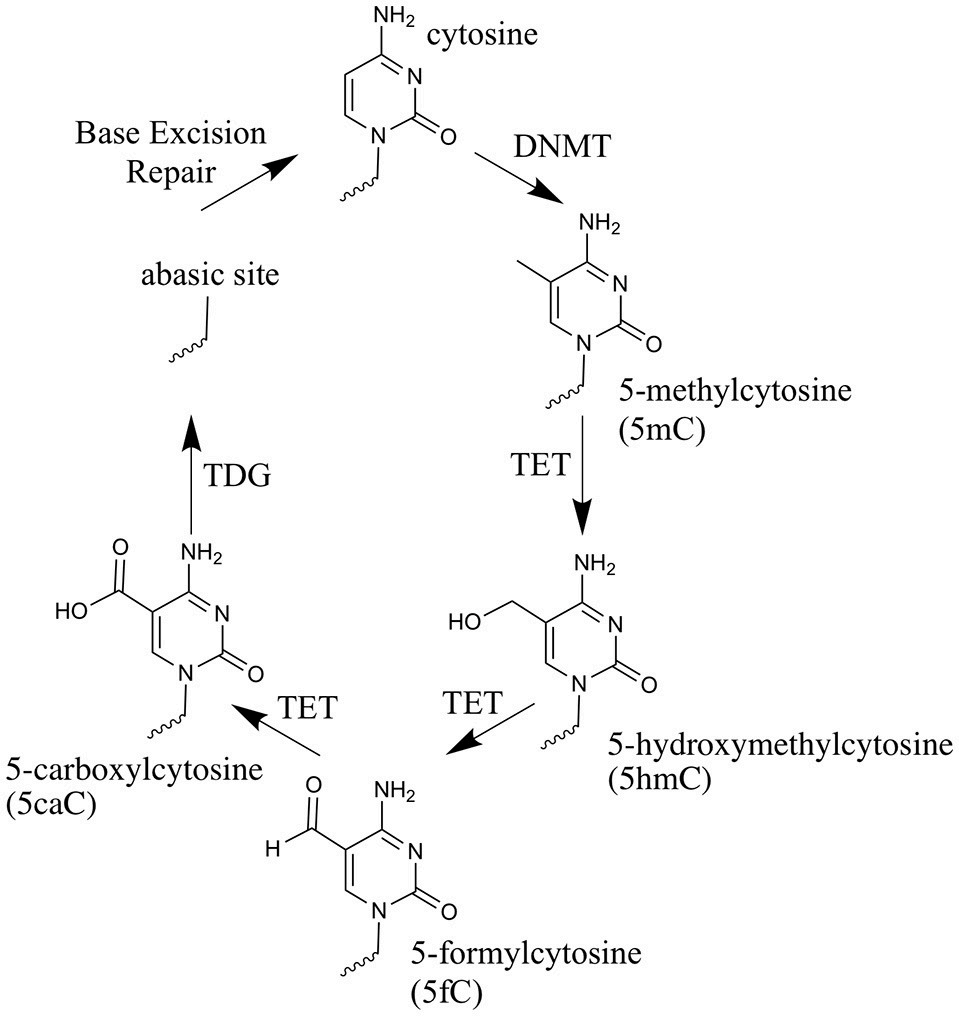
The cytosine oxidation cycle. The chemical structures of cytosine, 5-methylcytosine and each oxi-mC within DNA. Their synthesis route is depicted by arrows. The enzyme catalyzing each reaction and/or the physical process taking place, is labeled next to the arrow.

Over the past 8 years, renewed interest in active demethylation and the role of 5mC in development has been fueled by the discovery of a novel function for the ten-eleven translocation (TET) enzymes (Tahiliani et al., 2009; Ito et al., 2010). These enzymes catalyze the iterative oxidation of 5mC to: 5-hydroxymethylcytosine (5hmC), 5-formylcytosine (5fC) and 5-carboxylcytosine (5caC) (Kriaucionis and Heintz, 2009; Tahiliani et al., 2009; Ito et al., 2011), from here on collectively referred to as oxi-mCs. The discovery that Thymine DNA glycosylase (TDG) can excise 5fC and 5caC, leaving an abasic site to be re-filled by non-modified cytosine, allowed a pathway for TET-mediated active demethylation to be posited (He et al., 2011; Maiti and Drohat, 2011). Figure 1 illustrates the steps involved in active demethylation. A comprehensive discussion of currently proposed demethylation mechanisms is provided in the excellent review of Wu and Zhang (Wu and Zhang, 2017).

5hmC was first detected in viruses, using a combination of UV-spectroscopy and paper chromatography (Wyatt and Cohen, 1953), but the first reported detection of this modification in animals (Penn et al., 1972) could not be reproduced. In fact, the generation of 5hmC was interpreted as an artifact caused by the method for hydrolysing the DNA during sample preparation (Kothari and Shankar, 1976). Using thin-layer chromatography and the purified DNA from lysates of Purkinje neurons, the existence of 5hmC was proven in 2009 and it was shown to be a substantial component of mammalian genomic DNA (Kriaucionis and Heintz, 2009). This discovery was accompanied by the discovery that TET1 could oxidize 5mC to 5hmC (Tahiliani et al., 2009). Two other oxidized derivatives of 5mC; 5fC and 5caC were later identified (Ito et al., 2011).

Subsequent studies have since demonstrated the necessity for the pre-existence of 5mC for 5hmC formation. The knockdown of *Dnmt* expression results in the disappearance of 5hmC from the genome (Ficz et al., 2011; Williams et al., 2011). The direct generation of 5hmC from non-methylated DNA has not yet been reported, either *in vitro* or *in vivo* (Szwagierczak et al., 2010; Ficz et al., 2011).

Whilst participating as intermediates in an active demethylation pathway, evidence of a *bona fide* epigenetic function of oxi-mCs has also been presented. 5mC, 5hmC, 5fC, and 5caC all appear to have an individual set of binding proteins (or “readers”) that associate with them in a cell type-specific manner (Iurlaro et al., 2013; Spruijt et al., 2013). To show this, pulldowns of proteins crosslinked to DNA fragments specifically containing 5mC, 5hmC, 5fC, and 5caC on nuclear lysates from mouse embryonic stem cells (mESCs), mouse neuronal precursor cells (NPCs) and mouse adult brain cells were performed (Spruijt et al., 2013). Although the exclusion of indirectly bound factors from the analysis was not possible, the constituents of bound complexes were found to differ between both the oxi-5mCs and across some of the cell types analyzed in this study (Spruijt et al., 2013). Whilst most of the complex components were associated with DNA demethylation, indicative of oxi-mCs acting as intermediates in this process, transcription factors such as MeCP2, found to bind 5hmC, were also detected in these experiments (Spruijt et al., 2013). MeCP2 had previously been shown to interact with 5hmC in neuronal cells where 5hmC was mainly found associated with actively expressed genes (Mellén et al., 2012). This together with the finding that 5fC preferentially localizes to poised enhancers in mESCs suggests that oxidation of 5mC itself may serve a regulatory purpose (Song et al., 2013).

## Key regulators of mouse and zebrafish DNA methylation

In vertebrates, DNMT1 maintains existing methylation patterns (Stein et al., 1982; Yoder et al., 1997). There are three main splice variants of *Dnmt1* expressed during mouse development—*Dnmt1* (Li et al., 1992), the oocyte specific *Dnmt1o* and the somatic *Dnmt1s* (Howell et al., 2001; Cirio et al., 2008; Hirasawa et al., 2008). Expression of *Dnmt1* occurs after embryonic day 7 (E7) (Carlson et al., 1992; Mertineit et al., 1998). *Dnmt1o* is maternally deposited and the protein active in the zygote (Hirasawa et al., 2008). DNMT1s activity occurs primarily after the first cell division (Cirio et al., 2008), with limited activity likely in the zygote (Hirasawa et al., 2008). DNMT1 activity is believed to be guided by interaction with UHRF1 (also known as NP95) (Bostick et al., 2007; Sharif et al., 2007). UHRF1 also binds to H3K9me3, which marks heterochromatin (Bannister et al., 2001; Lachner et al., 2001; Peters et al., 2001), thus, UHRF1 directs DNMT1 to preserve the long-term silencing of genes (Fang et al., 2016; Harrison et al., 2016; Iurlaro et al., 2017). The *de novo* methylation of DNA is conducted by two enzymes in mice; DNMT3a and DNMT3b (Li et al., 1998; Okano et al., 1999). DNMT3a is first seen in the developing embryo at E7.5 (Okano et al., 1999), most strongly within extraembryonic tissue, with weak expression seen in the mesoderm (Okano et al., 1999). From E8.5, expression becomes ubiquitous (Okano et al., 1999). DNMT3b is first observed at E7.5 (Okano et al., 1999), most strongly within ectodermal tissue (Okano et al., 1999). DNMT3l is non-catalytic, but recruits DNMT3a and DNMT3b to nucleosomes devoid of H3K4 methylation (Saitou et al., 2012) and is expressed during gametogenesis (Bourc'his et al., 2001). Based upon sequence complementarity, zebrafish carry two likely orthologs to mouse *Dnmt3a*—*dnmt3aa* and *dnmt3ab*, which are conserved in other fish species (Goll and Halpern, 2011), with the orthologs to *Dnmt3b* likely being *dnmt3ba, dnmt3bb.1, dnmt3bb.2*, and *dnmt3bb.3* (Goll and Halpern, 2011). Orthologs to mouse *Dnmt1* and *Uhrf1* in zebrafish have also been identified (Mhanni et al., 2001; Goll and Halpern, 2011; Kent et al., 2016). Table 1 provides a list of the epigenetic players in mouse and zebrafish.

**Table 1:** Mouse and Zebrafish proteins involved in the cytosine methylation and oxidation pathway.

**Protein**	**Mouse gene**	**Mouse protein**	**Zebrafish gene**	**Zebrafish protein**
DNA methyltransferase 1	*Dnmt1*	Dnmt1	*dnmt1*	Dnmt1
		Dnmt1s		
		Dnmt1o		
DNA methyltransferase 3	*Dnmt3a*	Dnmt3a	*dnmt3aa*	Dnmt3aa
			*dnmt3ab*	Dnmt3ab
	*Dnmt3b*	Dnmt3b	*dnmt3ba*	Dnmt3ba
			*dnmt3bb.1*	Dnmt3bb.1
			*dnmt3bb.2*	Dnmt3bb.2
			*dnmt3bb.3*	Dnmt3bb.3
	*Dnmt3l*			
Ubiquitin-like with PHD and ring finger domains 1	*Uhrf1*	Uhrf1	*uhrf1*	Uhrf1
Tet methylcytosine dioxygenase 1	*Tet1*	Tet1	*tet1*	Tet1
Tet methylcytosine dioxygenase 2	*Tet2*	Tet2	*tet2*	Tet2
Tet methylcytosine dioxygenase 3	*Tet3*	Tet3	*tet3*	Tet3
Thymine DNA glycosylase	*Tdg*	Tdg	*tdg.1*	Tdg.1
			*tdg.2*	Tdg.2

In the following text, we will describe the early developmental differences in the dynamics of the methylome and hydroxymethylome in mice and zebrafish. The divergent phenotypes arising from the loss of DNMT, UHRF1 and TET in these two animals highlight the inter-species variation of the role for DNA methylation and hydroxymethylation during vertebrate embryogenesis.

## *Dnmt* and *Uhrf1* knockout phenotypes in mice

The homozygous knockout of *Dnmt1* causes lethality at E11 and these embryos display overall methylation levels of 30% compared to wildtype controls (Li et al., 1992) (A list of relevant mouse knockout phenotypes is given in Table 2). The presence of 5mC in these embryos was suggestive of other factors participating in its maintenance. These factors were subsequently identified to be DNMT1o (Mertineit et al., 1998) and DNMT1s (Howell et al., 2001). When DNMT1o deposition is prevented, embryonic imprints are largely maintained (Hirasawa et al., 2008), though embryos die at late gestation (Howell et al., 2001). This incomplete erasure is likely due to the presence of zygotic DNMT1s (Hirasawa et al., 2008). When DNMT1o is present in DNMT1s depleted embryos (Kurihara et al., 2008), maternal imprints are partially maintained (Kurihara et al., 2008). The methylome of the inactive maternal X-chromosome is protected by STELLA (Nakamura et al., 2007). The homozygous knockout of *Uhrf1* expression results in mid-gestational lethality (Sharif et al., 2007) that phenocopies the homozygous loss of *Dnmt1* (Li et al., 1992).

**Table 2:** Phenotypes resulting from the homozygous knockout of cytosine methylation and oxidation pathway genes in the mouse.

**Gene knockout**	**Homozygous KO phenotypes**	**References**
*Tet1*	Smaller size, reduced oocyte numbers—meiotic gene expression reduced	Dawlaty et al., 2011; Yamaguchi et al., 2012
*Tet2*	2–4 months—Increased white cell count	Li et al., 2011; Moran-Crusio et al., 2011
	Adult—predisposition to myeloid leukemia	
*Tet3*	Parental KO -> neo-natal lethality in heterozygous pups	Gu et al., 2011; Guo et al., 2014a; Inoue et al., 2015
*Tet1* + *Tet2*	Mid-gestational lethality of some embryos. Smaller ovaries and reduced fertility	Dawlaty et al., 2011
*Tet1* + *Tet2* + *Tet3*	Lethality at E6.5—gastrulation failure	Dai et al., 2016
*Tdg*	Lethality at E11.5—hemorrhage	Cortázar et al., 2011; Cortellino et al., 2011
*Dnmt1*	Lethality at E11	Li et al., 1992
*Dnmt1o*	Lethality at mid gestation. Imprinting largely maintained	Howell et al., 2001; Hirasawa et al., 2008
*Dnmt1s*	Non-lethal, partial loss of maternal imprints	Kurihara et al., 2008
*Uhrf1*	Lethality at E11, phenocopy of Dnmt1 KO	Okano et al., 1999; Sharif et al., 2007
*Dnmt3a*	Lethality—post-natal. Stunted growth, imprinting defects	Okano et al., 1999
*Dnmt3b*	Lethality—post-E9.5	Okano et al., 1999
*Dnmt3a* + *Dnmt3b*	Lethality—earlier than Dnmt3b	Okano et al., 1999

Mice heterozygous for either *Dnmt3a* or *Dnmt3b* knockout alleles are normal and fertile (Okano et al., 1999). The homozygous knockout of *Dnmt3a* is also not embryonically lethal, but results in imprinting defects, stunted growth and premature, post-natal death (Okano et al., 1999). *DNMT3b*^−/−^ mutant mice do not survive beyond mid-gestation and die after E9.5 (Okano et al., 1999). This timing is indicative of defects in extraembryonic tissue development (Copp, 1995). The combined knockout of *Dnmt3a* and *Dnmt3b* results in slightly earlier lethality than in DNMT3b-null mice (Okano et al., 1999).

In primordial germ cells (PGCs), DNMT1 is responsible for the maintenance of DNA methylation during their early maturation (Hargan-Calvopina et al., 2016). The germline-specific deletion of *Dnmt1* leads to premature meiotic gene expression and premature erasure of imprinting, causing infertility (Hargan-Calvopina et al., 2016). In PGCs, the loss of *Dnmt3l* blocks the re-establishment of methylation in maternally imprinted genes (Bourc'his et al., 2001). This loss results in the mid-gestational lethality of heterozygous progeny derived from outcrossing female mice that carry germline deletion of *Dnmt3l* (Bourc'his et al., 2001). These progeny display biallelic expression of maternally imprinted genes (Bourc'his et al., 2001), with the mid-gestational lethality being indicative of defects in placental formation (Copp, 1995). These *Dnmt3l* defects are re-capitulated in an analogous experiment, using females carrying the germline specific knockout of *Dnmt3a* instead (Kaneda et al., 2004). Curiously, the paternal germline-specific knockout of *Dnmt3a* also results in defects to gametogenesis, characterized by the absence of methylation at certain imprinted loci and sterility (Kaneda et al., 2004). DNMT3b appears to play no role in the development of the germline (Kaneda et al., 2004).

## Methods for studying methylomic and hydroxymethylomic changes

The three most common methods employed for detecting 5mC and oxi-mCs in mice are immunofluorescent staining, bisulfite sequencing and mass spectrometry, but each of these methodologies have limitations (reviewed in Wu and Zhang, 2017). Only mass spectrometry is capable of directly quantifying 5mC across the whole developmental span (Amouroux et al., 2016; Okamoto et al., 2016), but is not currently sensitive enough to quantify oxi-mCs during early development (Okamoto et al., 2016). In zebrafish, the large clutch size makes these problems less pronounced, with direct 5mC and 5hmC quantification being possible using mass spectrometry across the whole development span (Kamstra et al., 2015). In adult organisms, the study of inter-tissue 5mC and 5hmC variation is much more informative than overall changes (Kriaucionis and Heintz, 2009; Globisch et al., 2010; Kamstra et al., 2015). Fortunately, in adult tissue, quantification of 5mC and 5hmC is much easier than in embryos (Kriaucionis and Heintz, 2009; Globisch et al., 2010).

## The developmental profile of the mouse methylome

**Figure 2:**
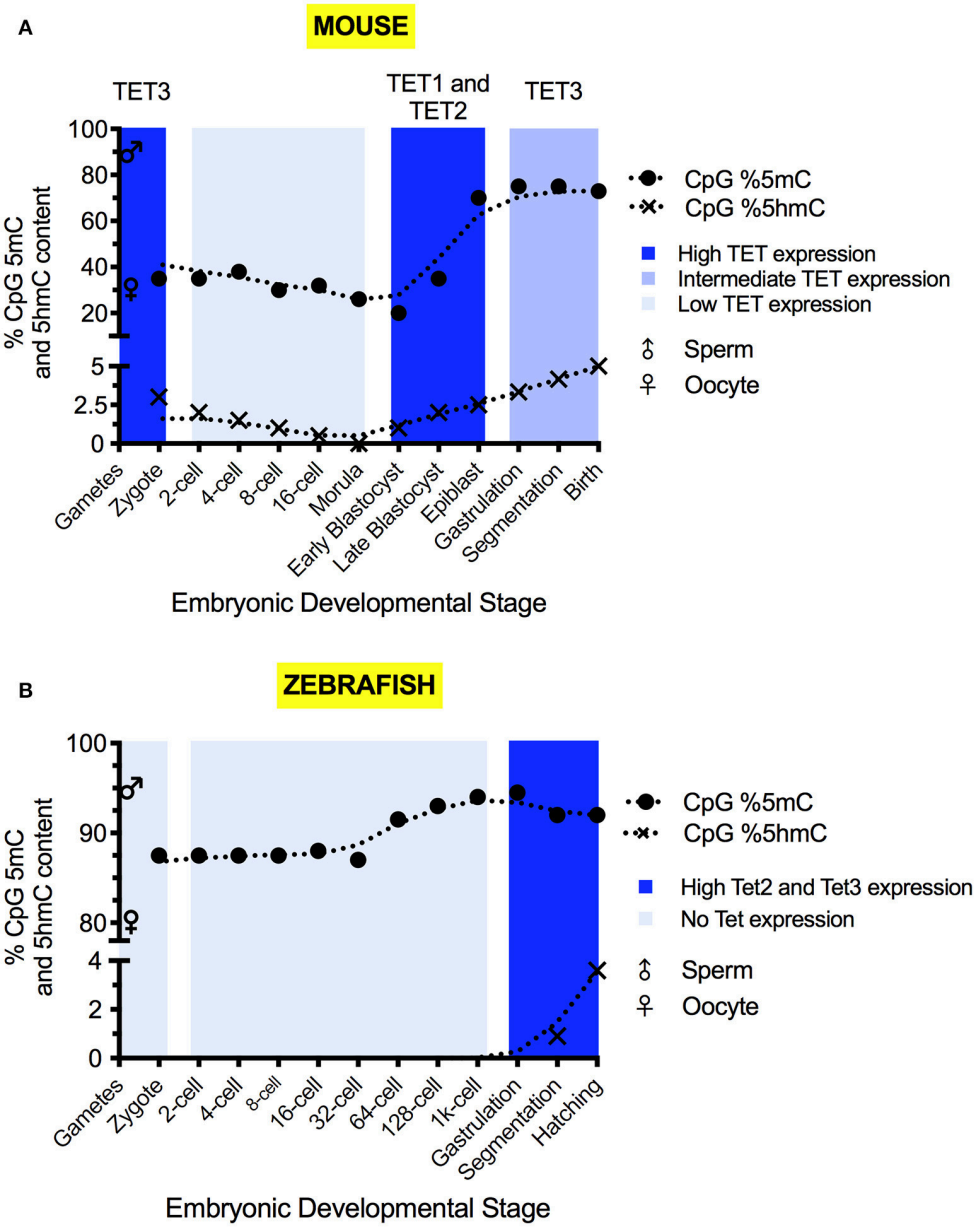
The 5mC and 5hmC content of the mouse **(A)** and zebrafish **(B)** during development. 5mC and 5hmC content are expressed as % of genomic CpG sites. Solid circles represent the overall genomic CpG 5mC status (Jiang et al., 2013; Amouroux et al., 2016; Okamoto et al., 2016; Guo et al., 2017), with crosses representing the overall genomic CpG 5hmC status (Pastor et al., 2013; Wu and Zhang, 2014; Kamstra et al., 2015; Amouroux et al., 2016). Sex symbols indicate the 5mC content of sperm or oocytes. Blue panels represent the general expression level of TET mRNA at particular stages (Yamaguchi et al., 2012; Ge et al., 2014; Amouroux et al., 2016; Bogdanović et al., 2016). X-axes labels indicate the developmental stage of the embryo, with shared positions being analogous between species.

The early developmental methylomic profile (i.e., pre-organogenesis) within mice, which is illustrated in Figure 2A, has been well defined and re-programming phases coincide with particular development stages (see these excellent reviews, Saitou et al., 2012; Iurlaro et al., 2017). Methylomic re-programming begins at fertilization. At this stage, the sperm and oocyte combine their respective haploid methylomes to form the zygotic methylome. Initial reprogramming of the parental pronuclei takes place rapidly and the first cell division then occurs (Gu et al., 2011; Guo et al., 2014a). Blastomeres divide and differentiation commences in the morula, from which the blastocyst forms. The blastocyst further differentiates into the epiblast and gastrulation occurs. Germline development begins with a population of epiblast cells being epigenetically specified to develop as PGCs (Lawson et al., 1999). Post-gastrulation, organogenesis begins and the embryo develops to adulthood.

Sperm carries a highly methylated genome, with 80–90% of CpG sites marked by 5mC (Amouroux et al., 2016; Guo et al., 2017). The oocyte is comparatively hypomethylated with approximately 30% of CpG sites marked (Amouroux et al., 2016; Guo et al., 2017). Discrepancies between the reported magnitude of methylomic changes exist due to differences of quantitation method used and the cellular pools analyzed (Amouroux et al., 2016; Guo et al., 2017). By the first cell division, paternal 5mC levels have declined to 30–40% of the sperm (Amouroux et al., 2016; Guo et al., 2017), whilst the maternal methylome remains relatively stable, with 5mC declining by no more than 10% (Okamoto et al., 2016; Guo et al., 2017). Overall zygotic methylation levels decrease by roughly 20% from fertilization to the first cell division (Guo et al., 2017) and, in addition to global demethylation of 5mC occurring, it has been shown that limited *de novo* methylation also takes place in the zygote (Amouroux et al., 2016). Between the 2-cell stage and 8-cell stage, minimal DNA demethylation takes place (Okamoto et al., 2016; Guo et al., 2017).

After the 8-cell stage, 5mC levels decline (Okamoto et al., 2016). Differentiation begins in the morula, with separation of the trophectoderm and the inner cell mass (ICM) allowing implantation to commence (Wang and Dey, 2006). In humans, the two cell layers are distinguished by slight differences to global 5mC levels (Guo et al., 2014b). Embryonic cells have lost their totipotency by blastocyst formation (Wang and Dey, 2006) and 5mC levels reach their developmental minimum outside of the germline (Smith et al., 2012; Kobayashi et al., 2013; Okamoto et al., 2016; Guo et al., 2017). Only 20% of blastocyst CpG sites are methylated (Okamoto et al., 2016; Guo et al., 2017), suggesting that low 5mC levels in mice are required for naïve pluripotency to enable both gastrulation and germline development.

The naïve pluripotent cells of the blastocyst's ICM differentiate into the epiblast in which re-methylation raises 5mC levels to 70% of CpG sites (Smith et al., 2012; Auclair et al., 2014). The epiblast generates the embryonic soma and the germline. PGC formation is epigenetically induced by a BMP signal released from the extraembryonic ectoderm (Lawson et al., 1999). The three embryonic germ layers arise as the primitive streak appears and marks the onset of gastrulation (Tam and Loebel, 2007). In somatic cells, 5mC continues to accumulate post-epiblast, and adult tissues typically display high levels of 5mC (Globisch et al., 2010).

Sexual reproduction requires the formation of gametes. These develop from PGCs. PGC formation begins at approximately E6.5, when BMP4 signaling from the extraembryonic ectoderm (Lawson et al., 1999) induces the transcription of *Blimp1* and *Prdm14* within a small number of epiblast cells (Saitou et al., 2012). These transcription factors repress the transcription of *Dnmt3a, Dnmt3b, Dnmt3l* and cause the repression of UHRF1 activity (Kagiwada et al., 2012; Iurlaro et al., 2017). The methylation dynamics of germline development have been well explored in this review (Messerschmidt et al., 2014). The re-establishment of totipotency in gametes requires erasure of genomic imprints (Hackett et al., 2013; Yamaguchi et al., 2013b), re-activation of the silenced X-chromosome (Chuva de Sousa Lopes et al., 2008) and reset of the somatic methylome within the PGC precursors (Saitou et al., 2012). This demethylation is complete prior to PGC sexual specification (Messerschmidt et al., 2014). In mice, germ cell specification and maturation is an epigenetic process, involving two main stages of demethylation (Seisenberger et al., 2012).

**Figure 3:**
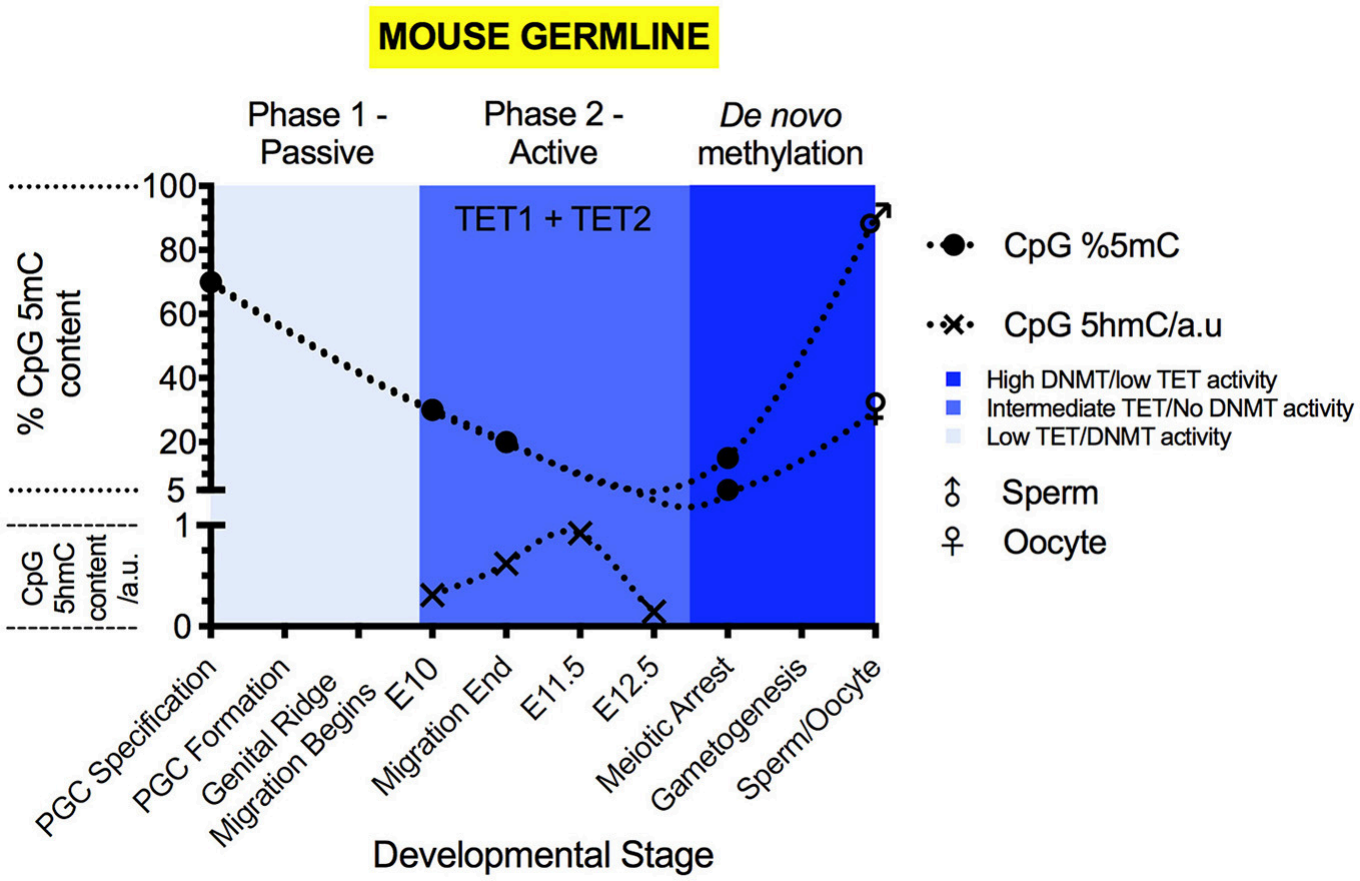
The 5mC and 5hmC content of the mouse germline during development. 5mC content is expressed as % of genomic CpG sites, while 5hmC is expressed as relative change to genomic 5hmC status. Solid circles represent the overall genomic CpG 5mC status (Seisenberger et al., 2012; Guo et al., 2017; Iurlaro et al., 2017), with crosses representing the relative genomic CpG 5hmC status (Hackett et al., 2013; Yamaguchi et al., 2013a). Sex symbols indicate the 5mC content of sperm or oocytes. Blue panels represent the general activities of TET and DNMT enzymes at particular stages (Kagiwada et al., 2012; Hackett et al., 2013). Above each colored panel, the coinciding methylation phase is labeled. X-axes labels indicate the developmental stage of the embryo.

The first stage of PGC demethylation commences upon their specification and results in a drop of 5mC levels from approximately 70% of CpG sites to 25% of CpG sites (Seisenberger et al., 2012) (please see Figure 3 for an illustration). This demethylation initiates the re-establishment of totipotency via the reversal of the somatic cell methylation patterns which had developed post-gastrulation (Seki et al., 2005; Kurimoto et al., 2008). During this stage DNMT1 maintains the methylation, specifically, of imprinted regions and meiotic genes (Hargan-Calvopina et al., 2016). DNMT1^−/−^ PGCs display premature expression of key meiotic factors for both sperm and oocyte development, such as *Stra8*, γ*H2AX*, and *Tex12*, as well as significantly reduced methylation at imprinting control regions (ICRs), for genes such as *H19* and *Snrpn* (Hargan-Calvopina et al., 2016).

Active demethylation and passive dilution of 5mC, localized to discrete genomic locations, drives the second phase of PGC methylomic reprogramming (E9.5-13.5) (Seisenberger et al., 2012; Yamaguchi et al., 2012; Hackett et al., 2013), after which totipotency is re-established. (Figure 3) The timing of demethylation during the second stage of reprogramming is not simultaneous at all sites (Hackett et al., 2013). Initiation between loci can differ by as much as 24 h (Hackett et al., 2013), suggesting that demethylation of imprints and genes involved in gametogenesis is progressive and controlled. However, areas of the genome are able to escape from the near complete erasure of 5mC in PGCs prior to their sexual specification and re-methylation (Lane et al., 2003; Borgel et al., 2010; Iurlaro et al., 2017). These “escapees” (Iurlaro et al., 2017) are typically repeat elements and of the IAPLTR1 subclass (Hackett et al., 2013), though evidence suggests that some parasitic elements are also demethylated (Wang et al., 2014), for currently obscure reasons.

Once demethylation is complete, sexual specification can proceed (Messerschmidt et al., 2014). In E13.5 female PGCs, the lowest level of 5mC content is observed, at 2.2% of CpG sites (Hackett et al., 2013). From the initiation of sexual specification onward, *de novo* methylation proceeds, leading to re-establishment of parental imprints in the gametes (Hata et al., 2002; Seisenberger et al., 2012). Eventually, sperm carry 5mC at 80–90% of CpG sites and oocytes carry 30% (Amouroux et al., 2016; Guo et al., 2017).

## The developmental profile of the mouse hydroxymethylome

5hmC is detectable at all stages of pre-implantational development (Ruzov et al., 2011) (Figure 2A). In the zygote, 5hmC is detectable at the mid-pronuclear stage (Ruzov et al., 2011; Amouroux et al., 2016) and increases in abundance up to the first cell division (Gu et al., 2011; Iqbal et al., 2011; Ruzov et al., 2011; Pastor et al., 2013; Amouroux et al., 2016). This increase accompanies the demethylation of the paternal genome seen over time in the zygote (Amouroux et al., 2016; Okamoto et al., 2016; Guo et al., 2017). Zygotic 5fC and 5caC formation transiently accompany 5hmC accumulation (Inoue et al., 2011; Wang et al., 2014; Amouroux et al., 2016). The embryonic levels of 5fC and 5caC are substantially reduced by the four-cell stage (Wang et al., 2014).

Changes to 5hmC levels after the first cell division are low until the morula forms, after which 5hmC content increases and peaks in the blastocyst ICM (Ruzov et al., 2011). As the ICM differentiates and gastrulation occurs, 5hmC becomes localized to particular tissue types and putative stem cell niches (Ruzov et al., 2011).

During PGC maturation, 5hmC is not present during the first phase of demethylation (Hackett et al., 2013; Yamaguchi et al., 2013a) (Figure 3). During the second phase of PGC demethylation, starting at E9.5, 5hmC becomes detectable (Hackett et al., 2013; Yamaguchi et al., 2013a) and the decrease in 5mC abundance is mirrored by increases to 5hmC levels. At E11.5, 5hmC content begins to decline progressively (Hackett et al., 2013; Yamaguchi et al., 2013a), reaching a minimum at E13.5 (Hackett et al., 2013; Yamaguchi et al., 2013a). During the second phase of demethylation in PGCs, 5hmC enrichment can be found at all genetic elements outside of the small number of constitutively methylated sites.

5hmC quantification in adult mice has been restricted to discrete tissues (Kriaucionis and Heintz, 2009; Globisch et al., 2010). LC-MS data from adult tissues reveals that 5hmC content is highly variable, ranging from approximately 0.35–0.7% of genomic cytosine within the CNS, particularly enriched in neurons (Kriaucionis and Heintz, 2009), to being practically undetectable in testes (Globisch et al., 2010). It is believed that neurons contain the highest amount of 5hmC within terminally differentiated cells (Globisch et al., 2010). Why this variation exists is unclear, but is suggestive of roles for 5hmC and TET in regulating the development of discrete adult tissue (Spruijt et al., 2013), or that oxi-mCs can operate as stable, *bona fide* epigenetic marks (Spruijt et al., 2013).

## *Tet* and *Tdg* mRNA expression patterns

*Tet3* mRNA is maternally deposited and protein is expressed in the zygote (Gu et al., 2011) (Figure 2A), before undergoing rapid degradation or cytoplasmic sequestration (Gu et al., 2011; Iqbal et al., 2011). *Tet3* mRNA is then re-expressed post mid-gestation (Yamaguchi et al., 2012). The zygotic expression of *Tet1* and *Tet2* mRNA is very low (Iqbal et al., 2011; Amouroux et al., 2016). Significant *Tet1* mRNA expression occurs after the zygotic genome activation (ZGA) and peaks in the ICM (Amouroux et al., 2016). Significant *Tet2* mRNA expression is mainly confined to the blastocyst and the somatic tissue post mid-gestation (Yamaguchi et al., 2012; Amouroux et al., 2016; Bogdanović et al., 2016).

In PGCs, *Tet1* mRNA expression peaks during the second phase of their methylomic reprogramming (Yamaguchi et al., 2012; Hackett et al., 2013) (Figure 3). PGCs display co-expression of *Tet1* and *Tet2* mRNA (Yamaguchi et al., 2012), however, *Tet2* mRNA expression peaks in the germline much later (Yamaguchi et al., 2012). The peak of *Tet2* mRNA expression coincides with the sudden onset of *Tet3* mRNA expression within oocytes (Gu et al., 2011; Yamaguchi et al., 2012).

During embryonic development, prior to the blastocyst, *Tdg* mRNA is barely detectable (Tang et al., 2011). In the ICM and, especially in mESCs, *Tdg* mRNA expression is comparatively high (Tang et al., 2011), while embryonic expression levels post-gastrulation have not been reported. *Tdg* mRNA is expressed at high levels within PGCs (Kagiwada et al., 2012) and is present from early to late stages of their maturation (Kagiwada et al., 2012).

## *Tet* and *Tdg* knockout phenotypes in mice

To study the role of TET1 in mouse development, chimeric males transplanted at blastocyst stage with *Tet1*^+/−^ mESCs were bred to WT females to produce *Tet1*^+/−^ progeny. These mice were phenotypically normal and fertile. Homozygous mice derived from a heterozygous incross, although smaller in size, turn out to be viable, showing that TET1 is not essential for ontogenesis (Table 2). When these mice are crossed to WT mice, however, litter sizes were smaller than expected, suggesting reduced fertility of *Tet1*^−/−^ males and females, and a role for TET1 in male and female germline development (Dawlaty et al., 2011).

An independently generated *Tet1* gene-trap line that displayed the same overall phenotype was used to further study the role of TET1 in the male and female germ line (Yamaguchi et al., 2012). Adult females homozygous for the gene trap allele had smaller ovaries with reduced germ cell numbers. Oocyte numbers were already reduced in the *Tet1*^−/−^ female fetus at E16.5. Fetal ovaries showed increased numbers of apoptotic cells that had failed to progress through the meiotic prophase. Analysis of the methylation profile of the female PGCs at E13.5 revealed that while genome wide demethylation was unaffected, a subset of meiotic genes displayed hypermethylation and reduced expression (Yamaguchi et al., 2012). Unlike the *Tet1* knockout ovaries, *Tet1* knockout testes were functionally normal. However, embryos derived from crosses of *Tet1*^−/−^ males with WT females showed heterogeneous phenotypes (Yamaguchi et al., 2013b). All displayed placental growth defects that resulted in variable embryonic and postnatal growth retardation. The placental defects were reminiscent of abnormalities observed in mice with a mutation in the imprinted gene *Peg10* (Ono et al., 2006). RNA-Seq experiments performed on E9.5 embryos demonstrated that imprinted genes were indeed dysregulated in the progeny (Yamaguchi et al., 2013b). In E13.5 PGCs of the *Tet1*^−/−^ homozygous males, imprinted genes displayed hypermethylation that is maintained in the sperm (Yamaguchi et al., 2013b). Altogether these data show that TET1 expression in developing PGCs is not required for global PGC genome demethylation, but plays an important role in (a) the activation of meiotic genes in the developing oocytes and (b) in the erasure of imprints in the germ cells.

*Tet2*^−/−^ mice display no gestational developmental defects, have normal morphology and are fertile (Li et al., 2011) (Table 2). Incrosses of *Tet2*^−/−^ mice produce normal litter sizes and morphologically normal embryos (Li et al., 2011). While heterozygous and homozygous *Tet2* mutants appear macroscopically normal, analysis of their blood at 2–4 months revealed increased numbers of white blood cells. In mice carrying hematopoietic stem cell (HSC)-specific depletion of *Tet2*, an increased rate of self-renewal, elevated myeloid progenitor proliferation and a predisposition to myeloid leukemia was observed (Moran-Crusio et al., 2011). These defects were cell-autonomous (Moran-Crusio et al., 2011). Interestingly, the phenotype of *Tet1* and *Tet2* double homozygous mice (Dawlaty et al., 2013) was reminiscent of *Tet1*^−/−^ homozygous mice (Yamaguchi et al., 2012, 2013b).

The expression of *Tet3* in oocytes and zygotes (Figure 2A) suggests a role for *Tet3* at the earliest stages of mouse embryonic development. To study this role, germline-specific knockouts were generated (Table 3). When female mice carrying the germline-specific *Tet3* knockout alleles were outcrossed to wildtype mice, the litter size was smaller than expected (Gu et al., 2011). In the zygote, 5mC levels were maintained and 5hmC levels failed to rise in the male and female pronuclei (Gu et al., 2011; Guo et al., 2014a). This coincided with a high rate of developmental failure at mid-gestation stages (Gu et al., 2011), which, however, since has been shown to be dependent on the genetic background (Inoue et al., 2015). Irrespective of this background, the *Tet3*^+/−^ progeny displayed a considerable degree of neonatal lethality, as well as growth defects which were also observed in *Tet3*^+/−^ mice that were derived from a cross of *Tet3*^+/−^ males with wildtype females, suggesting that the neonatal lethality is due to haploinsufficiency rather than the lack of 5mC oxidation at zygotic stages (Inoue et al., 2015). *Tet3* mutants all died neonatally (Gu et al., 2011). Altogether, these data suggest important roles for TET3 at different stages of embryonic development that require further investigation.

**Table 3:** Phenotypes resulting from the homozygous knockout of cytosine methylation and oxidation pathway genes in the mouse germline.

	**Homozygous KO phenotypes**	**References**
*Tet1*	Male and female germline -> smaller litter size when bred to WT	Dawlaty et al., 2011; Yamaguchi et al., 2012, 2013b
	Male germline -> heterozygous offspring display placental growth defects	
*Tet2*	No phenotype	Li et al., 2011; Dai et al., 2016
*Tet3*	No phenotype	Dai et al., 2016
*Dnmt1*	Infertility, premature meiotic gene expression and premature imprint erasure	Hargan-Calvopina et al., 2016
*Dnmt3a*	Female germline -> mid-gestational lethality of heterozygous offspring, inability to establish maternal imprints	Bourc'his et al., 2001; Kaneda et al., 2004
	Male germline -> gametogenic defects	
*Dnmt3b*	No phenotype	Kaneda et al., 2004
*Dnmt3l*	Phenocopy of Dnmt3a	Bourc'his et al., 2001

To investigate whether functional redundancy masked roles of TET proteins in embryonic development in the single and *Tet1*/*Tet2* double knockouts, triple knockout embryos were generated by deleting the three *Tet* genes in the maternal and paternal germline (Table 2). The mice carrying triple *Tet*^+/−^ sperm and oocytes were crossed to generate triple *Tet*^−/−^ progeny. These embryos initially developed normally, but then failed to progress beyond gastrulation, with a phenotype being observable at E6.5 (Dai et al., 2016). The phenotype consists of buckling of the epiblast layer in the primitive streak region, defective migration of mesodermal cells and a lack of head folds in the nascent fetus (Dai et al., 2016). In spite of these defects, the expression of early gastrulation markers is still seen (Dai et al., 2016). The definitive endoderm and axial mesoderm are specified, but fail to mature (Dai et al., 2016). The paraxial mesoderm is absent and neuroectodermal development is abnormal (Dai et al., 2016). These observations indicate that gastrulation was initiated, but had failed to progress (Dai et al., 2016). This phenotype was caused by hyperactive NODAL signaling due to the transcriptional silencing of *Lefty1* and *Lefty2* (Dai et al., 2016). The *Lefty* genes encode inhibitors of NODAL signaling (Meno et al., 1998, 1999). The silencing of the *Lefty* genes correlates with elevated DNA methylation at their cis-regulatory element in the absence of all three TET proteins (Dai et al., 2016). Normal *Lefty* expression and the phenotype of the embryos were rescued by the presence of a single copy of WT *Tet3* or by the knockout of *Dnmt3* (Dai et al., 2016).

TDG is essential for mouse embryonic development (Cortázar et al., 2011; Cortellino et al., 2011) (Table 2). While *Tdg*^+/−^ embryos and adult mice are normal, *Tdg*^−/−^ mice develop internal hemorrhage and necrosis (Cortázar et al., 2011; Cortellino et al., 2011). The phenotype manifests at E10.5 and leads to lethality at E11.5, for unknown reasons (Wu and Zhang, 2017). The analysis of gene expression in mouse embryonic fibroblasts (MEFs) isolated from E9.5 *Tdg*^−/−^ mouse embryos suggests a role for TDG in transcriptional gene regulation (Cortázar et al., 2011; Cortellino et al., 2011). *Tdg*^−/−^ MEFs, as well as *Tdg*^−/−^ ES cells induced to differentiate into neural progenitor cells show reduced expression of numerous developmental genes (Cortázar et al., 2011). The downregulation of these genes in differentiating cells coincides with the presence of repressive histone modifications and the methylation of CpG sites in their promoter regions (Cortázar et al., 2011; Cortellino et al., 2011). These data suggest that TDG acts in concert with other partners like the TET proteins, the deaminase AID and histone modifiers, like CBP/p300 and MLL to maintain activating marks on developmental gene promoters to prevent their Polycomb- and DNMT3a/b-mediated repression during differentiation (Cortázar et al., 2011; Cortellino et al., 2011).

## Zebrafish embryonic development and the role of DNA methylation

In zebrafish, external fertilization of the egg is followed by external embryonic development. The embryo's yolk provides the nutrition and gas diffusion through the skin is sufficient for gas exchange. Extraembryonic membranes are not formed, and the embryos undergo an extended period of cell cleavages before zygotic genome activation occurs at approximately the 1,000 (1k) cell stage (Kimmel et al., 1995; Pálfy et al., 2017). Unlike in mice, PGCs are not induced, but predetermined by the maternal deposition of germ plasm in the oocyte (Raz, 2003). Therefore, blastodermal cells of the 1k stage embryo that lack germ plasm do not need to be programmed to be able give rise to cells of the germline. Thus, naïve pluripotent cells do not exist. These differences in embryology are reflected in the methylome and hydroxymethylome of zebrafish, as will be explained below.

DNA methylation plays an important role during zebrafish development. The loss of zygotic expression of Dnmt1 in *dnmt1*^−/−^ mutants causes an embryonic phenotype that manifests from 84 hpf and leads to larval death by 8 dpf. Embryos exhibit a small-sized pancreas, liver and eyes, as well as dysmorphic branchial arches (Anderson et al., 2009). Within the exocrine pancreas, acinar cells are specified, but fail to proliferate and eventually undergo apoptosis (Anderson et al., 2009). A smaller liver is also observed upon zygotic loss of *uhrf1* expression (Sadler et al., 2007; Jacob et al., 2015). *uhrf1*^−/−^ mutants show defects in liver outgrowth and liver regeneration. Hepatocytes undergo re-replication of their DNA, fail to divide and undergo apoptosis. *Uhrf1* and *dnmt1* mutants also display defects in lens development (Tittle et al., 2011) and intestinal barrier function (Marjoram et al., 2015). *Dnmt1* mutants also struggle to maintain haematopoietic progenitor cells (Liu et al., 2015). Maternal supply of Dnmt1 and Uhrf1 is likely to mask earlier roles of DNA methylation in the zygotic mutants.

Translation-blocking morpholinos that target maternal and zygotic mRNA show that depletion of maternal and zygotic Uhrf1 protein causes pre-gastrulation lethality, suggesting an essential role for maternal Uhrf1 protein at ZGA (Chu et al., 2012; Kent et al., 2016). By contrast, Dnmt1 morphants display late differentiation defects in the intestine, the exocrine pancreas and the retina (Rai et al., 2006). The presence of 6 orthologs of mouse *Dnmt3* in zebrafish makes loss of function studies difficult as the role of individual genes may be masked by functional redundancy (Goll and Halpern, 2011). Nevertheless, *dnmt3bb.1* mutants have been shown to have very specific defects in haematopoietic progenitor maintenance (Gore et al., 2016). Morpholino-mediated knockdown of Dnmt3bb.2 causes neurogenic defects in the brain and the retina (Rai et al., 2010). Inhibition of all Dnmt activity, using 5-azacytidine, lead to defects in somite development, notochord and muscle, as well as to trunk shortening (Martin et al., 1999; Goll and Halpern, 2011), however the compound's instability in water complicates data analysis and interpretation (Goll and Halpern, 2011). The different embryonic phenotypes caused by 5-azacytidine treatment and Uhrf1, Dnmt1 *and* Dnmt3 morpholino-injection highlights that more work is required to gain a better understanding of the role of DNA methylation during early zebrafish embryogenesis.

## Developmental dynamics of the zebrafish methylome and hydroxymethylome

Bisulfite sequencing of genomic DNA isolated from sperm, oocytes and early stage embryos revealed that 91–95% of CpG dinucleotides are methylated in sperm and 75–80% are methylated in oocytes (Jiang et al., 2013; Potok et al., 2013) (Figure 2B). There is very little methylation in non-CpG sites (Potok et al., 2013). As the zygote undergoes cell divisions, DNA methylation is reduced and reaches a minimum at the 64-cell stage. Subsequent re-methylation is gradual and takes CpG methylation levels back to that of sperm by the time the embryo reaches sphere stage (Potok et al., 2013). By the time of ZGA, the oocyte methylome has been reprogrammed to match the sperm (Jiang et al., 2013; Potok et al., 2013). Close inspection of the methylation in CpG islands at promoter sites revealed that the methylome is static for large numbers of genes (Potok et al., 2013). Constitutively hypomethylated TSSs are found in genes involved in basal cellular processes, such as metabolism, transcription and early development (Potok et al., 2013). Shared, constitutively hypermethylated promoters are associated with genes related to later developmental processes, such as neurogenesis (Potok et al., 2013). A small number of CpG islands display dynamic methylation patterns (Potok et al., 2013). A subset of those are hypermethylated in the maternal genome and are subsequently demethylated prior to the midblastula transition (MBT) (Potok et al., 2013). These include the TSSs of genes with roles in germ cell and embryonic development (Potok et al., 2013). Other genes have hypomethylated TSSs in the oocyte that are methylated upon zygotic gene activation. These include genes involved in oogenesis and late development (Potok et al., 2013). Comparison of methylomic and transcriptomic data revealed that genes with hypermethylated promoter sequences were silent while genes with hypomethylated TSSs were more likely to be expressed. Some hypomethylated genes that are not expressed are likely to be under the additional epigenetic control, for example by histones (Jiang et al., 2013; Potok et al., 2013). The *hox* genes are hypomethylated in sperm, but packaged in H3K4me3 and H3K27me3-bivalently marked chromatin and, therefore, poised, but not active (Jiang et al., 2013; Potok et al., 2013). Outside of promoters, differentially methylated regions are also found insides genes. These are suspected to include enhancer sequences (Potok et al., 2013). Thus, the dynamic methylomic changes that occur on the maternal genome adjust the maternal genome to the paternal genome. This is in preparation for the onset of biallelic zygotic gene transcription. The methylomic changes on the maternal genome do not require the presence of the paternal template. They occur even in maternal haploids generated by parthenogenesis, when eggs are triggered to develop by fertilization with UV-treated and therefore genome-inactivated sperm (Potok et al., 2013). The absence of widespread demethylation of the zebrafish genome between fertilization and ZGA likely reflects differences in embryonic development compared to mice. The early zebrafish cells neither need to give rise to extraembryonic tissue nor do they need to generate PGCs. The lack of imprinting (Macleod et al., 1999; Potok et al., 2013) means that the global, active demethylation occurring prior to gametogenesis in mice is not required in zebrafish (Yamaguchi et al., 2013b). PGCs are also pre-determined by the inheritance of germ plasm and set aside at the beginning of embryonic development (Raz, 2003). Thus, blastomeres lacking germ plasm neither require the ability to generate PGCs nor need to be programmed for naïve pluripotency, which is linked to genome demethylation. This allows the zebrafish to pre-set the zygotic methylome in the gametes, with minor amendments to the maternal methylome being performed during the early cleavage period.

From MBT onwards, the embryonic methylome begins to diverge from the sperm (Jiang et al., 2013). The methylomic changes that occur during this period are much more substantial than those seen prior to MBT (Jiang et al., 2013; Potok et al., 2013). A comparison of the methylome of sphere stage embryos with that of differentiated muscle cells reveals thousands of differentially methylated sequences. Most of these are likely to be intronic enhancers and many of them are hypermethylated in the differentiated muscle cells (Potok et al., 2013). Single-base resolution methylome maps of zebrafish embryos at different stages of development also pinpoint thousands of differentially methylated regions of the genome (Lee et al., 2015). Many of these lie outside of promoters, CpG islands and nearby shores. These sequences are enriched for evolutionary conserved sequences or have previously been associated with histone modification and transcription factor binding signatures, suggesting that they constitute cis-regulatory elements. Twenty of the elements have been tested in stable transgenic zebrafish and shown to act as tissue-specific enhancers (Lee et al., 2015). Thus, differential methylation is another indicator for active enhancer sequences. Overall, the use of DNA methylation in the regulation of promoter and enhancer activity is conserved in fish and mouse.

The changes in the methylation profiles seen after MBT coincide with an increase in 5hmC levels (Lee et al., 2015) (Figure 2B)—indicative of active demethylation occurring. 5hmC only becomes observable at 12 hpf (Kamstra et al., 2015) and, unlike in mice, 5hmC does not correlate positively with pluripotency (Ruzov et al., 2011; Almeida et al., 2012). Embryonic 5hmC content peaks at 96 hpf with 0.23% of genomic cytosine, and the rise is concomitant with a decline in 5mC content (Kamstra et al., 2015). 5hmC presence is maintained into adulthood, displaying inter-tissue enrichment variation akin to mammals (Globisch et al., 2010; Kamstra et al., 2015). As in mammals (Globisch et al., 2010), 5hmC is most abundant in the brain, reaching 0.5% of cytosine content (Kamstra et al., 2015) and the reasons for this enrichment are likewise unknown.

## Expression profiles of *Tet* and *Tdg* mRNA and knockout phenotypes in zebrafish

The zebrafish genome contains paralogs to *Tdg* in the mouse, these are *tdg.1* and *tdg.2* (Yates et al., 2016, ENSEMBL release 90). Their expression patterns have not been published. Neither loss of function nor gain of function data has been published.

The expression of the *tet* genes and the presence of 5hmC are virtually undetectable prior to 24 hpf (Almeida et al., 2012; Ge et al., 2014; Kamstra et al., 2015; Bogdanović et al., 2016), suggesting they make no contribution to pre-organogenic zebrafish development. The generally low expression of *tet1* mRNA compared to *tet2* and *tet3* (Bogdanović et al., 2016) indicates that Tet2 and Tet3 make the dominant contribution to 5mC oxidation in zebrafish. Why the level of *tet1* expression is low remains to be determined.

Individual *tet* gene deletion does not cause a noticeable reduction to 5hmC levels at embryonic stages. All *tet* mutants are viable and fertile (Li et al., 2015) (Table 4). The lack of 5hmC reduction in zebrafish suggests functional redundancy and that compensatory increases in expression to the remaining *tet* genes may occur. The latter has not been explored. In double *tet2*^−/−^ *tet3*^−/−^ fish, a reduction in the 5hmC level is observed at 5 dpf and embryonic abnormalities are visible (Li et al., 2015). The phenotype includes the loss of HSC formation on day 1 (Li et al., 2015). From day 1 of development, abnormalities in eye development, brain morphology and pigmentation are visible, and defects in HSC production are detectable (Li et al., 2015). The *tet2*^−/−^ and *tet3*^−/−^ double knockout zebrafish die during the larval period (Li et al., 2015). Triple *tet* mutants show a further reduction of 5mC levels, but present the same phenotype (Li et al., 2015). There are no gastrulation defects. Thus, the role that mouse Tet proteins have in the activation of *lefty1* expression and the control of the Nodal morphogen gradient prior to gastrulation does not seem to be conserved in the zebrafish embryo. By contrast, a late role for Tet2 in haematopoietic cells is conserved from fish to man. 24-month-old *Tet2* mutant zebrafish display a myelodysplastic syndrome that is also observed in human patients and mouse models with TET2 mutation in their haematopoietic cells (Gjini et al., 2015). Altogether, the phenotypes of the *tet2* single and *tet2/tet3* double mutant zebrafish suggest that Tet proteins play very specific roles during lineage specification and differentiation in zebrafish. Current data suggest that these roles may or may not be conserved between mammals and fish.

**Table 4:** Phenotypes resulting from the loss of function of cytosine methylation and oxidation pathway genes in the zebrafish.

**Gene knockout**	**Homozygous KO phenotype**	**References**
*tet1*	No phenotype	Li et al., 2015
*tet2*	Adults—myelodysplastic syndrome susceptibility	Gjini et al., 2015
*tet3*	No phenotype	Li et al., 2015
*tet1* + *tet2*	No phenotype in embryo	Li et al., 2015
*tet1* + *tet3*	No phenotype in embryos	Li et al., 2015
*tet2* + *tet3*	Lethality—larval period. Reduction of HSC formation. Defects in eye development, brain morphology and pigmentation	Li et al., 2015
*tet1* + *tet2* + *tet3*	Phenocopy of tet2 + tet3	Li et al., 2015
*tdg.1* + *tdg.2*	Unknown	
*dnmt1*	Lethality at 8dpf. Small-sized exocrine pancreas, liver and eyes, lens defects, defects in intestinal barrier function and haematopoietic progenitor maintenance	Anderson et al., 2009; Tittle et al., 2011; Liu et al., 2015
*dnmt3bb.1*	Defects in haematopoietic progenitor maintenance	Gore et al., 2016
*uhrf1*	Defects in liver outgrowth and regeneration, lens defects, defects in intestinal barrier function and haematopoietic progenitor maintenance	Sadler et al., 2007; Jacob et al., 2015
Gene knockdown	Morphant Phenotype	
*uhrf1*	Lethality—pre-gastrulation	Chu et al., 2012; Kent et al., 2016
*dnmt3bb.2*	Defects in brain and retinal neurogenesis	Rai et al., 2010
Effect	5-azacytidine Treatment Phenotype	
DNA demethylation	Defects in somite and notochord development. Trunk shortening	Martin et al., 1999; Goll and Halpern, 2011

## Concluding remarks

During vertebrate embryonic development, cellular differentiation relies on the differential expression of thousands of genes. Epigenetic mechanisms, including DNA methylation, act on cis-regulatory elements to control their expression. DNA methylation usually leads to the repression of gene expression. Zebrafish and mouse use essentially the same enzymatic machinery to set, maintain, modify and remove these methylation marks. The way how they use this machinery early in embryogenesis, however, differs considerably and reflects important differences in their embryogenesis. The mammalian embryo (a) has the immediate need to generate extraembryonic membranes for its survival, (b) uses imprinting to prevent parthenogenesis, and (c) needs to generate naïve pluripotent cells in the blastocyst that are able give rise to cells of the germline. Zebrafish do not have any of these requirements. Their yolk provides the nutrition, while gas exchange is achieved through the skin, as embryos develop externally. Zebrafish have not evolved a mechanism to prevent parthenogenesis through imprinting. Furthermore, zebrafish PGCs are predetermined by germ-plasm and are not induced through epigenetic mechanisms. Therefore, unlike in the mouse, the roles of zebrafish Tet proteins are restricted to the regulation of specific processes occurring during lineage specification and differentiation. The few examples we know suggest that their involvement in a particular process may or may not be conserved between mammals and fish.

## Author contributions

All authors listed, have made substantial, direct and intellectual contribution to the work, and approved it for publication.

### Conflict of interest statement

The authors declare that the research was conducted in the absence of any commercial or financial relationships that could be construed as a potential conflict of interest.
